# Improving the efficiency of homologous recombination by chemical and biological approaches in *Yarrowia lipolytica*

**DOI:** 10.1371/journal.pone.0194954

**Published:** 2018-03-22

**Authors:** In-Seung Jang, Byung Jo Yu, Ji Yeon Jang, Jonggeon Jegal, Ju Young Lee

**Affiliations:** 1 Center for Bio-based Chemistry, Korea Research Institute of Chemical Technology (KRICT), Jongga-ro, Jung-gu, Ulsan, Republic of Korea; 2 Intelligent Sustainable Materials R&D Group, Research Institute of Sustainable Manufacturing System, Korea Institute of Industrial Technology (KITECH), Yandaegiro-gil, Ipjang-myeon, Seobuk-gu, Cheonan-si, Chungcheongnam-do, Republic of Korea; CNR, ITALY

## Abstract

Gene targeting is a challenge in *Yarrowia lipolytica* (*Y*. *lipolytica*) where non-homologous end-joining (NHEJ) is predominant over homologous recombination (HR). To improve the frequency and efficiency of HR in *Y*. *lipolytica*, the *ku70* gene responsible for a double stand break (DSB) repair in the NHEJ pathway was disrupted, and the cell cycle was synchronized to the S-phase with hydroxyurea, respectively. Consequently, the HR frequency was over 46% with very short homology regions (50 bp): the *pex10* gene was accurately deleted at a frequency of 60% and the β-carotene biosynthetic genes were integrated at the correct locus at an average frequency of 53%. For repeated use, the URA3 marker gene was also excised and deleted at a frequency of 100% by HR between the 100 bp homology regions flanking the URA3 gene. It was shown that appropriate combination of these chemical and biological approaches was very effective to promote HR and construct genetically modified *Y*. *lipolytica* strains for biotechnological applications.

## Introduction

The oleaginous yeast *Yarrowia lipolytica* (*Y*. *lipolytica*) is an attractive host with multiple biotechnological and industrial applications for production of various chemicals and heterologous proteins (i.e. proteases, lipases and RNase) [1–3]. This is due to its ability to produce and accumulate large quantities of lipids [4–6]), utilize and grow on hydrophobic carbon sources [7, 8], and secrete native and heterologous proteins at high levels [9–11]. The availability of the genome sequence of *Y*. *lipolytica* [12, 13] and development of genetic tools such as transformation methods [14–16] for gene deletion [17] and integration [18–23] also enable the redesign of a synthetic metabolic pathway [24]. Recently, CRISPR-Cas9-based genome engineering tools have been successfully developed for marker-less gene disruption and integration in *Y*. *lipolytica* [25], and are now available for engineering metabolic pathways [26]. Despite the availability of several genetic tools for the manipulation of *Y*. *lipolytica*, homologous integration of exogenous DNA can be cumbersome when a short stretch of homologous sequence is used.

Integration of a DNA fragment into a genome requires the action of a double-strand break (DSB) repair [27, 28]. DSB can be repaired through the two major pathways: homologous recombination (HR) and non-homologous end-joining (NHEJ). These two repair pathways act independently and are considered to function competitively [29]. NHEJ causes random integration. In contrast, HR results in targeted integration at a homologous locus [30]. In *Y*. *lipolytica*, NHEJ is dominant over HR and hence integration of exogenous linear DNA fragment occurs at random in the genome. Depending on the transformation method, HR only occurs at acceptable rates (>80%) with 0.5 to 1 Kb of homolog arm to the gene of interest [17]. A principal component of the NHEJ pathway is the KU70/KU80 heterodimer, comprising of an evolutionary conserved pair of proteins in all eukaryotes. For the last 10 years, efforts to improve gene targeting efficiency have focused on abolishing the NHEJ pathway by disrupting either *ku70* or *ku80* gene [31, 32]. Furthermore, by disrupting the *ku70* gene, the HR frequency is also dramatically increased in *Y*. *lipolytica* [31, 33].

In addition, HR and NHEJ repair DSB generated during S/G2 and G1 phase of the cell cycle in *S*. *cerevisiae*, respectively [34]. Thus increase in the S-phase population is critical for efficient gene targeting by HR [35]. Hydroxyurea (HU), a potent inhibitor of ribonucleotide reductase [36–38], lowers the level of dNTPs and stops DNA synthesis and arrests replication. HU is commonly used to synchronize growing cells in the S-phase of the cell cycle and has been shown to induce homologous recombination in various yeast strains including *Y*. *lipolytica* [35]. Here we have studied the effects of HU-mediated cell cycle synchronization in the *ku70*-disrupted *Y*. *lipolytica* strain, both to improve the frequency of HR and to develop methods for enhancing its process. These methods greatly facilitate HR and offer a convenient and efficient technique for rapid one-step PCR targeted gene replacement.

## Materials and methods

### Yeast stains and growth media

All the strains used in this study were constructed in the *Y*. *lipolytica* PO1f background (ATCC MYA-2613), a leucine and uracil auxotroph devoid of any secreted protease activity [39]. The *ku70*-disrupted *Y*. *lipolytica* strain was constructed using the *ku70* deletion cassettes with URA3 marker as described by Verbeke [31]. All the strains used in this study are listed in Table 1.

**Table 1:** Strains and plasmids used in this study.

Strain or plasmid	Description	Source or reference
*Strains*		
Po1f	*MATa*, *leu2-270*, *ura3-302*, *xpr2-322*, *axp-2*	ATCC [39]
Δku70	Po1f *ku70Δ*	This study
Δpex10 URA3	Po1f *pex10Δ* :: *ura3*	This study
Δku70 Δpex10 URA3	Po1f *ku70Δ pex10Δ* :: *ura3*	This study
crtI URA3	Po1f *ku70Δ pox1Δ* :: *crtI ura3*	This study
crtI	Po1f *ku70Δ pox1Δ* :: *crtI*	This study
crtI/YB URA3	Po1f *ku70Δ pox1Δ* :: *crI pox2Δ* :: *crtYB ura3*	This study
crtI/YB	Po1f *ku70Δ pox1Δ* :: *crI pox2Δ* :: *crtYB*	This study
crtI/YB/E URA3	Po1f *ku70Δ pox1Δ* :: *crtI pox2Δ* :: *crtYB pox3Δ* :: *crtE ura3*	This study
crtI/YB/E	Po1f *ku70Δ pox1Δ* :: *crtI pox2Δ* :: *crtYB pox3Δ* :: *crtE*	This study
*Plasmids*		
pUC57-URA3	Plasmid containing 3HA-*URA3*-3HA deletion cassette	[40]
pYLEX1	JMP62-*LEU*	Yeastern^a^
pUC-ylURA3	Plasmid containing 3HA-*ylURA3*-3HA deletion cassette	This study
pUC-ylURA3-P_4UAS1B_	Plasmid containing P_4UAS1B_, T_XPR2_, 3HA-*ylURA3*-3HA integration cassette	This study
pUC-ylURA3-crtI	*crtI* ORF cloned between the PmlI and KpnI site of pUC-*ylURA3*-P_4UAS1B_	This study
pUC-ylURA3-crtYB	*crtYB* ORF cloned between the PmlI and KpnI site of pUC-*ylURA3*-P_4UAS1B_	This study
pUC-ylURA3-crtE	*crtE* ORF cloned between the PmlI and KpnI site of pUC-*ylURA3*-P_4UAS1B_	This study

^a^The pYLEX1 plasmid was purchased from Yeastern Biotech Company (Taipei, Taiwan).

Media and growth conditions for *Escherichia coli* and *Y*. *lipolytica* have been previously described by Sambrook and Russell [41], and Barth and Gaillardin [42], respectively. While, the Yeast Extract Peptone Dextrose (YPD) medium was prepared with 20 g/L bacto peptone (Difco Laboratories), 10 g/L yeast extract (Difco), 20 g/L glucose (Sigma-Aldrich), the Yeast Nitrogen Base (YNB) medium was made with 6.7 g/L yeast nitrogen base (without amino acids) (Difco), 0.69 g/L complete amino acid supplement mixture (CSM)-uracil supplement (MP Biomedicals), and 20 g/L glucose. HU was obtained from Sigma-Aldrich.

### Construction of a URA3-blaster cassette for PCR-based gene targeting

To delete genes from the *Y*. *lipolytica* genome, the pUC-ylURA3 plasmid was constructed by inserting the recyclable 3HA-*URA3*-3HA URA-blaster cassette consisting of the *Y*. *lipolytica* URA3 gene flanked by 100 bp 3HA (a three-tandem repeat of the HA tag) direct repeats into the pUC57 plasmid (GeneScript, Piscataway, NJ) [40].

To integrate the target genes into the genome of *Y*. *lipolytica*, the P_4UAS1B_-T_XPR2_ fragment from pYLEX1 (Yeastern Biotech Company, Taipei, Taiwan) was cloned into pUC-ylURA3, generating the pUC-ylURA3-P_4UAS1B_ plasmid. Subsequently, to integrate the β-carotene producing pathway into the genome of *Y*. *lipolytica*, the sequences of *crtI*, *crtYB*, and *crtE* genes from *Xanthophyllomyces dendrorhous* were codon optimized and cloned into the pUC-ylURA3-P_4UAS1B_ plasmid to produce pUC-ylURA3-crtI, pUC-ylURA3-crtYB, and pUC-ylURA3-crtE plasmids, respectively.

### Transformation of *Y*. *lipolytica*

*Y*. *lipolytica* strain was incubated in YPD medium at 30°C with shaking at 250 rpm. After overnight incubation, the cells were transferred into 25 mL of YPD medium to give an initial OD_600_ of 0.5. After further incubation for 3 h at 30°C with constant shaking at 250 rpm, 50 mM HU was added, and the *Y*. *lipolytica* culture was incubated for an additional 2 h [35]. Subsequently, the cells were washed twice with 10 mL of sterile dd-H_2_O and resuspended at 5 x 10^8^ cells per mL in 0.1 M lithium acetate. An aliquot of 100 μL of the cell suspension in a sterile microcentrifuge tube was centrifuged and the supernatant was discarded. To the cell pellet 5 μL of URA3-blaster DNA cassette, 102.5 μL of the transformation cocktail (90 μL of 50% poly(ethylene glycol) 4000, 5 μL of 2 M dithiothreitol, 5 μL of 2 M lithium acetate pH 6.0, and 2.5 μL of single-stranded carried DNA (10 μg/μL)) were added. The cell pellet/transformation cocktail mixture was mixed well by vortexing and heat shocked at 39°C for 1 h. The cells were then harvested by centrifugation, the pellet was resuspended in 1 mL of YPD medium and incubated overnight at 30°C. Approximately 200 μL of the cell suspension was spread on a yeast selective plate (YSC-URA medium with 2% (v/v) glucose: synthetic complex medium lacking uracil, 2% (v/v) glucose) and incubated for several days at 30°C.

### Gene deletion and integration

To delete the *pex10* gene from the *Y*. *lipolytica* genome, the deletion cassette with 50 bp of homology arm to the *pex10* gene was amplified from pUC-ylURA3 plasmid by polymerase chain reaction (PCR), and the amplified *pex10* gene deletion cassette was then introduced into *ku70*-disrupted *Y*. *lipolytica*, producing the Δ*pex10* strain.

The β-carotene-producing strain was constructed by first replacing the *pox1* gene with the *crtI* gene under the control of the 4UAS1B promoter. The *P*_*4UAS1B*_*-crtI* replacement cassette with 50 bp of homology arm to the *pox1* gene was amplified by PCR from pUC-ylURA3-crtI and introduced into *ku70*-disrupted *Y*. *lipolytica*. The *pox2* gene was then replaced by the *P*_*4UAS1B*_*-crtYB* DNA fragment in the *pox1Δ* :: *P*_*4UAS1B*_*-crtI* background by the same method. Subsequently, the *pox3* gene was replaced by the *P*_*4UAS1B*_*-crtE* DNA fragment in the *pox1Δ* :: *P*_*4UAS1B*_*-crtI pox2Δ* :: *P*_*4UAS1B*_*-crtYB* background, producing crtI/YB/E strain (*pox1Δ* :: *P*_*4UAS1B*_*-crtI pox2Δ* :: *P*_*4UAS1B*_*-crtYB pox3Δ* :: *P*_*4UAS1B*_*-crtE)*.

At each step of the strain constructions, the modifications to each target region were verified by PCR, using pairs of primers that flanked the endpoints of the respective target regions.

### A 5-Fluoroorotic acid selection for URA3 marker reuse

A 5 mL of 5-fluoroortic acid (5-FOA) solution (100 mg/mL, Zymo Research, USA) in dimethyl sulfoxide and a 5 ml of uracil solution (2 g/L) were added to 500 mL of minimal medium agar plates (synthetic complex medium lacking uracil, 2% (v/v) glucose) [31]. The cells grown overnight in the YPD liquid medium were spread onto the 5-FOA plates and incubated for several days at 30°C.

### Production of β-carotene by flask fermentation

The β-carotene-producing strain was grown in 5 mL of YPD medium. After overnight incubation at 30°C with constant shaking at 250 rpm, the cells were harvested by centrifugation and transferred into 50 mL of YPD medium, to give an initial OD_600_ of 0.5. Subsequently, the fermentation was run for 6 days at 30°C and 250 rpm.

### Analysis of β-carotene

The cells were harvested by centrifugation, resuspended in 0.7 mL of dimethyl sulfoxide, and then incubated for 10 min at 55°C followed by 45°C for 15 min after an equal volume of acetone was added to the suspension [43]. After centrifugation, the supernatants containing β-carotene were analyzed by high-performance liquid chromatography (HPLC; Agilent Technologies Series 1260 Infinity system, Agilent, USA) at 450 nm. A Kinetex C18 column (150 mm X 4.6 mm, 5 μm pore size; Phenomenex, Torrance, CA) was used for sample separation. Methanol, acetonitrile, and dichloromethane (42:42:16) were used in the mobile phase at a flow rate of 1.0 mL/min at 30°C.

The molecular mass of β-carotene was analyzed by Synapt G2 HDMS quadrupole time-of-flight (TOF) mass spectrometer equipped with an electrospray ion source (Waters, Milford, MA, USA). After calibrating the instrument with NaF solution, the sample was dissolved in 100% MeOH and introduced by direct infusion at a flow rate of 20 μL/min into the ion source operating in positive mode. All spectra within the range of 100 to 1000 m/z were acquired. Leucine enkephalin was used as the lock mass for the exact mass measurement correction.

## Results and discussion

### Construction of a URA3-Blaster cassette for PCR-based gene targeting and marker reuse

**Fig 1:**
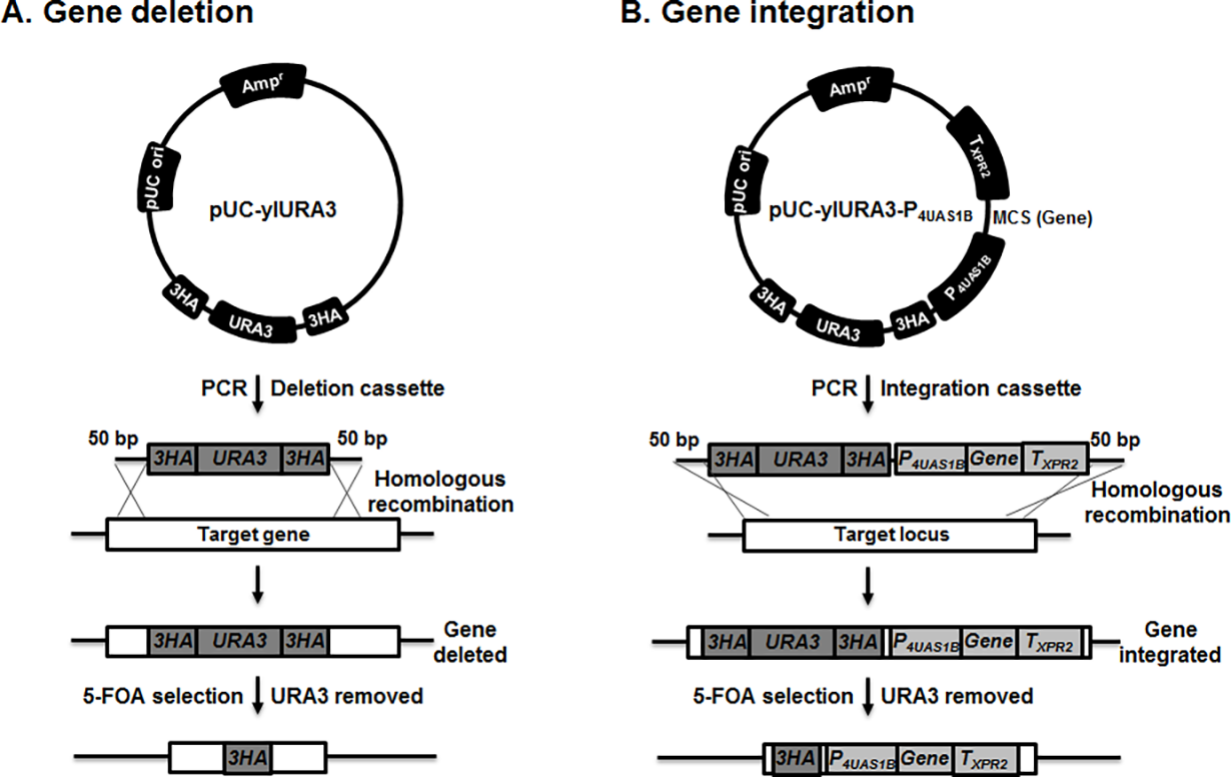
Schematic view of PCR-based gene targeting and URA3 marker reuse in *Y*. *lipolytica*. The URA3-blaster cassette with 50 bp of homology arm to target gene or locus was amplified from pUC-ylURA3 or pUC-ylURA3-P_4UAS1B_ plasmid. The URA3-blaster cassettes are integrated into the *Y*. *lipolytica* genome by HR and then HR between the 100 bp 3HA direct repeat results in excision of the URA3 marker at the integrated locus. (A) Gene deletion cassettes amplified from the pUC-ylURA3 plasmid. (B) Gene integration cassettes amplified the pUC-ylURA3-P_4UAS1B_ plasmid.

In *Y*. *lipolytica*, few methods for gene targeting have been reported, and all of them are laborious and time-consuming. Therefore, we designed a URA3-blaster cassette for an efficient PCR-based gene targeting for the *Y*. *lipolytica* strain, using short primers (70 bp) to flank both sides of the URA3 marker gene that were 50 bp homology arm to the target gene (Fig 1) that is based on the *S*. *cerevisiae* URA3-blaster system [40, 44]. The URA3-blaster cassette allows the reuse of the auxotrophic *Y*. *lipolytica* URA3 selection marker. A 3HA*-URA3-*3HA cassette (consisting of a *Y*. *lipolytica* URA3 gene flanked by 100 bp 3HA direct repeats) was integrated into the *Y*. *lipolytica* genome to either disrupt or delete a gene (Fig 1A). A P_4UAS1B_-T_XPR2_-3HA*-URA3-*3HA expression cassette is introduced into the *Y*. *lipolytica* genome and expressed a heterologous gene of interest (Fig 1B). This expression cassette consists of a 4UAS1B promoter upstream of a heterologous gene of interest, a downstream XPR2 transcription terminator and a 3HA*-URA3-*3HA cassette. Gene targeting is achieved by the HR between 50 bp of gene-specific regions flanking the cassette and the 50 bp homologous regions of the gene of interest in the genome. Subsequent intrachromosomal homologous recombination between the 100 bp 3HA direct repeats flanking the URA3 gene in the cassette leads to the loss of the URA3 marker, and the selection for the URA^-^ revertant in 5-FOA-containing medium.

### Efficiency of gene targeting and URA3 marker deletion by HR

To examine the effect of S-phase arrest on gene targeting, actively dividing wild-type and the *ku70*-disrupted *Y*. *lipolytica* strains were grown in the presence or absence of HU prior to transformation. Cells treated or untreated with HU were transformed with a *pex10*-deletion cassette with 50 bp of homology arm to the *pex10* gene. The *pex10* gene (the peroxisomal biogenesis gene) was selected, because it is often disrupted during engineering for high lipid accumulation in *Y*. *lipolytica*. In a previous work, the deletion frequency of *pex10* gene was 4.5% with a 50 bp homology arm, and 91% with a 1 kb homology arm in the *ku70*-disrupted *Y*. *lipolytica* strain [33]. As shown in Table 2, the efficiency of gene targeting was significantly higher in HU-treated cells (wild-type and the *ku70*-disrupted *Y*. *lipolytica*) than in untreated cells. The highest frequency for *pex10* deletion (90%) was observed in the HU-treated wild-type *Y*. *lipolytica* strain and the lowest (0%) in the untreated wild-type and *ku70*-disrupted *Y*. *lipolytica* strains, thus confirming the contribution of S-phase arrest to gene targeting. Indeed, recent work by *Tsakraklides et al*. showed that S-phase arrest by HU serves to facilitate the gene targeting of short homologous region (37–50 bp) in wild-type *Y*. *lipolytica* strain [35].

**Table 2:** Efficiency of gene deletion. Cells treated or untreated with HU were transformed with a *pex10*-deletion cassette with 50 bp of homology arm to the *pex10* gene. The *pex10* deletion rates (%) are shown and the number of total transformants screened is included in parentheses. WT indicates the wild-type *Y*. *lipolytica* Po1f strain. The experiments were performed in duplicate.

Strains	Target gene	Targeting homology length	Transformation condition	
			Untreated cells % gene targeting (total transformants screened)	HU-treated cells % gene targeting (total transformants screened)
WT	*pex10*	50 bp	0% (20)	90% (20)
Δku70			0% (20)	60% (35)

For URA3 marker reuse, we next examined efficiency of URA3 marker deletion by HR using the above constructed *pex10*-deleted strains: the *pex10*-deleted wild-type (*Δpex10*) and the *pex10*-deleted *ku70*-disrupted (*Δku70 Δpex10*) strains. The overall efficiency of URA3 marker deletion by HR was 0% in the *Δpex10* strain and 100% in the *Δku70 Δpex10* strain (Table 3). The URA3 marker gene is successfully excised and deleted by intrachromosomal HR between the 100 bp of 3HA homology regions flanking the URA3 gene in the *ku70*-disrupted strain. Therefore, *ku70* disruption allows repetitive rounds of transformations by the URA3 marker reuse for sequential and multiple genetic modifications. All gene deletions were verified by PCR (data not shown).

**Table 3:** Efficiency of URA3 marker deletion by HR. For URA3 selection marker reuse, the strains integrated with deletion or integration cassette containing URA3 marker were grown overnight in the YPD liquid medium and then plated on the 5-FOA selection medium. The percentage of URA3 marker deletion is shown and the number of total colonies screened is included in parentheses. The experiments were performed in duplicate.

Strains	% URA3 marker deletion by HR (total colonies screened)
Δpex10 URA3	No colonies
Δku70 Δpex10 URA3	100% (10)
crtI URA3	100% (10)
crtI/YB URA3	100% (10)
crtI/YB/E URA3	100% (10)

Combined, the increased frequency of HR by HU-mediated S-phase arrest and *ku70* disruption demonstrates an efficient tool for gene targeting and marker reuse in *Y*. *lipolytica*. This system was thus used as a gene targeting tool for further study.

### Integration of β-carotene biosynthetic genes

**Fig 2:**
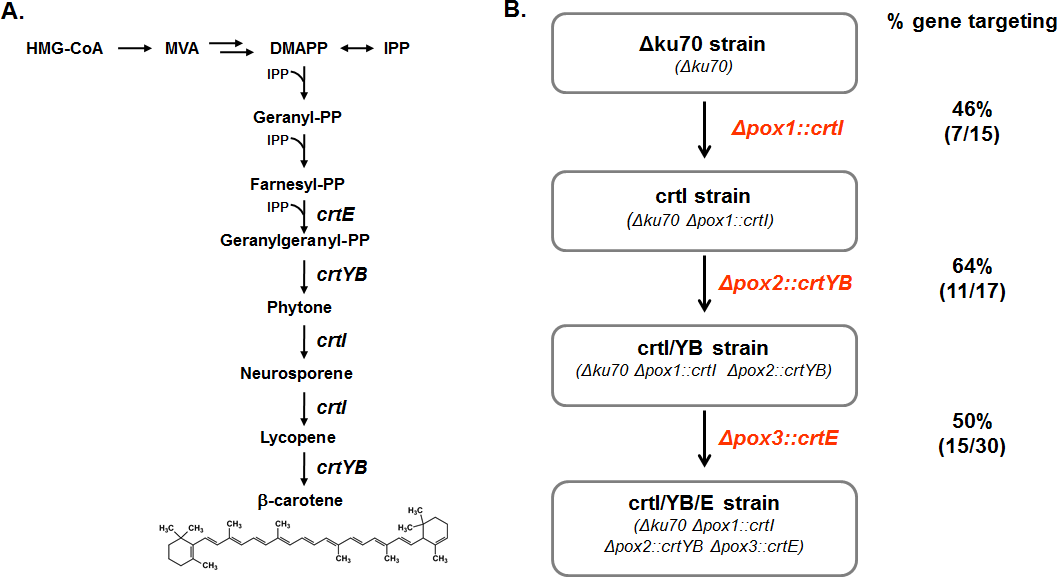
Engineering of β-carotene biosynthetic pathway. (A) A schematic representation of the β-carotene biosynthetic pathway in *Y*. *lipolytica*. Integrated genes include geranyl diphosphate synthase (*crtE*), phytoene synthase (*crtYB*) and carotene desaturase (*crtI*). (B) Scheme for the construction of the β-carotene producing strain and the efficiency of targeted gene integration. The *crtI*, *crtYB* and *crtE* genes, driven by their individual 4UAS1B promoters, were integrated into the *pox1*, *pox2* and *pox3* sites in the *ku70*-disrupted *Y*. *lipolytica* strain, respectively. Cells treated with HU were transformed with the gene replacement cassette with 50 bp of homology arm to the target gene. The gene targeting rate (%) is shown, and the numbers in parentheses represent the correct integrants/total transformants screened. The experiments were performed in duplicate. HMG-CoA, 3-hydroxy-3-methylgluratyl-coenzyme A; MVA, mevalonic acid; DMPAA, dimethylallyl pyrophosphate; IPP, isopentyl pyrophosphate; PP, pyrophosphate.

**Fig 3:**
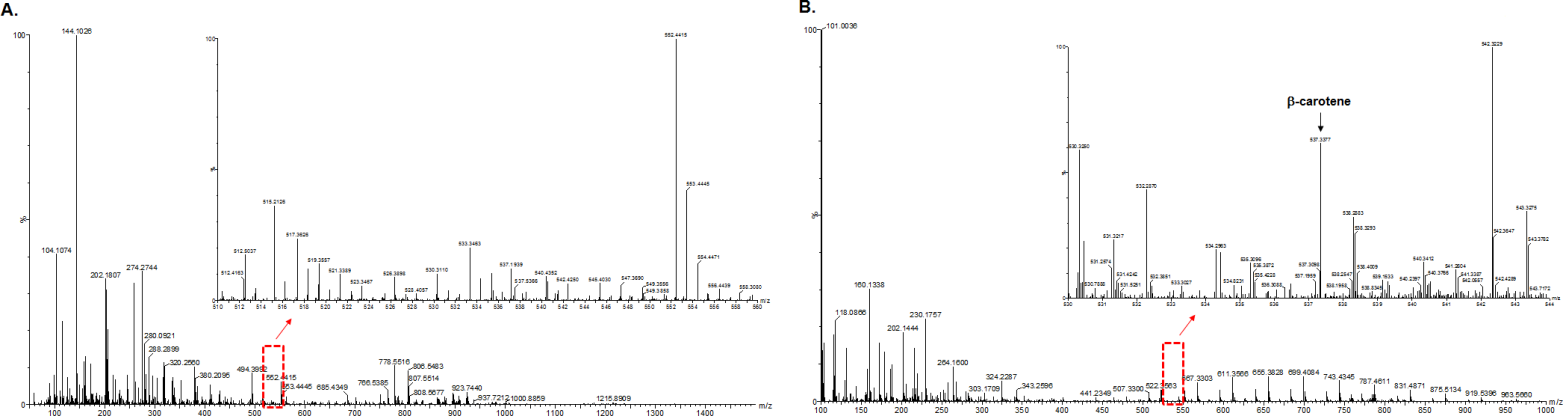
ESI-mass spectrum and representative expansion of β-carotene produced by the resulting β-carotene producing strain, crtI/YB/E. (A) ESI-mass spectrum of the wild-type *Y*. *lipolytica* strain. (B) ESI-mass spectrum of the resulting β–carotene producing strain (crtI/YB/E). All strains was cultivated for 6 days at 30°C in 50 mL of YPD medium containing 20 g/L glucose. The experiments were performed in duplicate, and the representative results are shown.

A key challenge in constructing a synthetic metabolic pathway in microbial hosts is to efficiently perform iterative modifications of multiple gene integrations and deletions. Therefore, we next demonstrated the application of this system by integrating 3 different β-carotene biosynthetic genes at different loci in the *ku70*-disrupted *Y*. *lipolytica* strain. The β-carotene biosynthetic pathway includes carotenogenic genes *crtE* (geranyl diphosphate synthase), *crtYB* (phytoene synthase) and *crtI* (carotene desaturase) from *Xanthophyllomyces dendrorhous*, whose expression enabled the synthesis of β-carotene in yeast (Fig 2A) [45, 46]. *Y*. *lipolytica* codon-optimized *crtI*, *crtYB* and *crtE* each driven by a 4UAS1B promoter were integrated into the *pox1*, *pox2* and *pox3* sites, respectively. The *pox1*, *pox2* and *pox3* genes (the β-oxidation genes) were selected because they are often disrupted during engineering for high lipid accumulation. At each step of strain construction, the modifications at each target region were verified by PCR, using pairs of primers that flanked the endpoints of the respective target regions (data not shown). The frequency of correct integration was 46% for *crtI*, 64% for *crtYB* and 50% for *crtE* (Fig 2B). The resulting β-carotene producing strain, crtI/YB/E produced 1.47 mg/L of β-carotene when cells were grown in flask (Fig 3).

Taken together, in this study, gene targeting (46–64%) was achieved with very short homology regions (50 bp) to the target locus in *Y*. *lipolytica*, which was as effective as in *S*. *cerevisiae*, where the gene targeting efficiency ranged from 17–60% with 35–51 bp of homology regions [47]. Short homology regions were part of the oligonucleotide primers used to amplify the gene deletion or integration cassette containing the URA3 selectable marker, obviating the need to construct gene targeting vectors with long homology regions, as in conventional gene targeting methods. Our method provides an easy-to-use tool for rapid strain development and reduces the associated costs. Therefore, this gene targeting system is an efficient platform for sequential and multiple genetic modifications in *Y*. *lipolytica*.
